# Spinal neurons require Islet1 for subtype-specific differentiation of electrical excitability

**DOI:** 10.1186/1749-8104-9-19

**Published:** 2014-08-22

**Authors:** Rosa L Moreno, Angeles B Ribera

**Affiliations:** 1 Department of Physiology, University of Colorado Anschutz Medical Campus, RC-1 North, 7403A, Mailstop 8307, 12800 E 19th Ave., 80045 Aurora, CO, USA

**Keywords:** Islet1, Zebrafish, Spinal Cord, Motor Neuron, Sensory Neuron, Electrical Excitability

## Abstract

**Background:**

In the spinal cord, stereotypic patterns of transcription factor expression uniquely identify neuronal subtypes. These transcription factors function combinatorially to regulate gene expression. Consequently, a single transcription factor may regulate divergent development programs by participation in different combinatorial codes. One such factor, the LIM-homeodomain transcription factor Islet1, is expressed in the vertebrate spinal cord. In mouse, chick and zebrafish, motor and sensory neurons require Islet1 for specification of biochemical and morphological signatures. Little is known, however, about the role that Islet1 might play for development of electrical membrane properties in vertebrates. Here we test for a role of Islet1 in differentiation of excitable membrane properties of zebrafish spinal neurons.

**Results:**

We focus our studies on the role of Islet1 in two populations of early born zebrafish spinal neurons: ventral caudal primary motor neurons (CaPs) and dorsal sensory Rohon-Beard cells (RBs). We take advantage of transgenic lines that express green fluorescent protein (GFP) to identify CaPs, RBs and several classes of interneurons for electrophysiological study. Upon knock-down of Islet1, cells occupying CaP-like and RB-like positions continue to express GFP. With respect to voltage-dependent currents, CaP-like and RB-like neurons have novel repertoires that distinguish them from control CaPs and RBs, and, in some respects, resemble those of neighboring interneurons. The action potentials fired by CaP-like and RB-like neurons also have significantly different properties compared to those elicited from control CaPs and RBs.

**Conclusions:**

Overall, our findings suggest that, for both ventral motor and dorsal sensory neurons, Islet1 directs differentiation programs that ultimately specify electrical membrane as well as morphological properties that act together to sculpt neuron identity.

## Background

Results of molecular and morphological studies of spinal neurons support the view that homeodomain (HD) transcription factors orchestrate genetic programs that specify neuronal identity (for review, see [1-5]). In invertebrates and chordates, HD transcription factors also play a role in specification of neuronal electrical membrane properties that also reflect a neuron’s identity [6-8]. Whether HD transcription factors act similarly in specifying electrical properties of vertebrate neurons is not known.

Islet1 is a HD transcription factor belonging to the Lin/Isl/Mec-like (LIM) conserved zinc finger domain class [9-11]. In vertebrates, motor neurons require Islet1 for their determination, survival and subsequent subtype specification [12-16]. In addition, Islet1 plays a role in specification of mammalian sensory neuron subtypes [17].

In zebrafish, consistent with mammalian and avian studies, motor neurons express *isl1*[18-20] and Islet1 knock-down leads to a loss of motor neurons [13,14]. On the basis of morphological and molecular properties, cells within the motor neuron progenitor domain differentiate but lack essential motor neuron-like molecular and morphological signatures, such as peripherally projecting axons [13,14]. In the dorsal cord, Islet1 knock-down effects have been examined by studying Rohon-Beard cells (RBs), a population of *isl1*-expressing early born primary sensory neurons that have large somas, extend central as well as peripheral processes, and express stereotypic molecular markers [18-25]. Following blockade of Islet1 function, dorsal neurons are present that occupy RB-like positions, have large somas, extend central axons, and express RB molecular markers [21,26,27]. Such results have led to the view that Islet1 is dispensable for RB but not motor neuron fate. However, the majority of these dorsal neurons fail to extend peripheral processes, an essential morphological hallmark of primary sensory neurons.

We assay effects of Islet1 knock-down on excitable membrane properties, sensitive measures of neuronal identity and subtype [7,8,28,29]. We find that, upon Islet1 knock-down, both dorsal and ventral neurons that occupy the stereotypical positions of RB and motor neurons have novel electrical properties that distinguish them from sensory or motor neurons, respectively, of control embryos. These results support the view that, for both motor and sensory neurons, Islet1 plays a role in the genetic programs that specify their membrane currents and thus regulate multiple properties that sculpt a neuronal identity.

## Results

### Spinal neurons with large somas persist after knock-down of Islet1

We first examined the effects of Islet1 knock-down on spinal sensory and motor neuron number and morphology. We used several transgenic lines that express green fluorescent protein (GFP) in specific neuronal subtypes. Furthermore, the early born sensory and motor neurons that we study here have large, stereotypically positioned somas and axons with characteristic morphologies [22,23,30-33].

**Figure 1:**
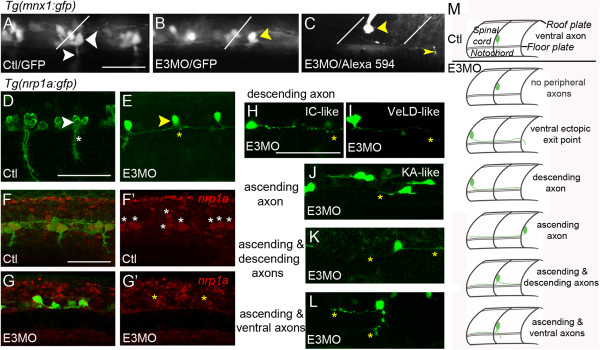
**Caudal primary motor neuron-like neurons have abnormal axonal trajectories that resemble those of interneurons.** In all figures, unless indicated otherwise, images present lateral views of embryos (rostral left, dorsal up). **(A)** In control (Ctl) *Tg(mnx1:gfp)ml2* 22 to 26 hours post-fertilization (hpf) embryos, primary motor neurons (PMNs) express green fluorescent protein (GFP). The caudal PMN (CaP; large white arrowhead) projects its peripheral axon (small white arrowhead) ventrally. White lines **(A, B** and **C)** indicate somite boundaries. **(B)** In *Tg(mnx1:gfp)ml2* E3 morphants, GFP^+^ cells persist in the ventral spinal cord. GFP^+^ CaP-like cells (yellow arrowhead) have somas in CaP positions but project axons centrally rather than peripherally. **(C)** In another *Tg(mnx1:gfp)ml2* E3 morphant, a GFP^+^ CaP-like cell (yellow arrowhead), filled with Alexa 594, has an axon that extends caudally to exit in the neighboring hemisegment (thin yellow arrowhead). **(D-L) (D)** In uninjected *Tg(nrp1a:gfp)js12* embryos, CaP (white arrowhead) has its soma immediately dorsal to the motor axon exit point (white asterisk). **(E)** E3 morphants have few GFP^+^ ventral neurons. A CaP-like cell (yellow arrowhead) lacks a peripheral axon. **(F**, **F’)** GFP^+^ PMNs of control *Tg(nrp1a:gfp)js12* embryos (**F’**, white asterisks) express *nrp1a*. **(G**, **G’)** Following Islet1 knock-down, few *nrp1a*/GFP^+^ (**G’**, yellow asterisks) neurons are present. **(H-L)** In E3 morphants, many GFP^+^ neurons have axons that bypass normal exit points and extend centrally either caudally (**H** and **I**), rostrally **(J)** or in both directions **(K)**. Occasionally, a GFP^+^ CaP-like cell extends a peripheral as well as a central axon **(L)**. **(M)** The top cartoon depicts control CaP axon morphology, and the six lower cartoons exemplify the range of CaP-like axonal phenotypes revealed by either dye filling or confocal analysis of *Tg(nrp1a:gfp)js12* E3 morphants. Scale bars = 50 μm in **A** (for **A** to **C**), **D** (for **D** and **E**), **F** (for **F** to **G**’) and **H** (for **H** to **L**). IC, ipsilateral commissural; KA, Kolmer-Agduhr; VeLD, ventral lateral.

For study of primary motor neurons (PMNs), we used the *Tg(nrp1a:gfp)js12* and *Tg(mnx1:gfp)ml2* lines, in which motor neurons express GFP [29,34,35]. At 1 day post-fertilization, control *Tg(nrp1a:gfp)js12* and *Tg(mnx1:gfp)ml2* embryos express GFP in PMNs (Figure 1). As reported previously [36], E3 morphant *Tg(mnx1:gfp)ml2* embryos maintain GFP expression in ventral spinal cord neurons that occupy PMN positions (Figure 1A,B). However, after Islet1 knock-down, ventral GFP^+^ cells have no or few peripherally projecting axons that exit in the same hemisegment as the soma (Figure 1B,C). In the *Tg(nrp1a:gfp)js12* line, however, Islet1 knock-down leads to loss of GFP expression and only a few ventral spinal neurons express the reporter (Figure 1D,E). Concomitant with the loss of GFP, RNA *in situ* hybridization reveals less *nrp1a* mRNA expression in E3 morphants (Figure 1F-G’). The persistence of GFP expression in the *Tg(mnx1:gfp)ml2* but not the *Tg(nrp1a:gfp)js12* line suggests that a novel neuronal population develops instead of PMNs.

At 24 hours post-fertilization (hpf), control PMNs have a prominent axon that projects to the periphery but no significant central ones. Hutchinson and Eisen [13] found that Islet1 knock-down results in few or no peripherally projecting motor axons. We obtained similar results indicating that Islet1 knock-down alters the development of neurons with somas in the PMN location (Figure 1A-E and H-M). We focus here on the PMN known as caudal primary motor neurons (CaP). In E3 morphants, we refer to neurons with somas in the characteristic CaP position as CaP-like cells.

We examined CaP-like axonal morphologies in E3 morphants in detail by taking advantage of the sparse GFP expression in the *Tg(nrp1a:gfp)js12* line and also by dye filling individual cells via patch pipets (for example, Figure 1C). Within single embryos, CaP-like cells display a range of novel axonal morphologies (Figure 1H-M, Table 1). Several CaP-like axons do not project to the periphery but rather resemble the central projections of Kolmer-Agduhr (KA’ and KA”), ventral lateral descending (VeLD) and ipsilateral caudal (IC) interneurons [13,22,23,30,37]. Overall, the effects of Islet1 knock-down on ventral spinal neurons support the view that a novel population of neurons develops upon knock-down of Islet1.

**Table 1:** Novel axonal morphologies of CaP-like cells

**Axonal morphology**	**Frequency**
Peripheral, truncated	26%^*2*^
Peripheral, exit point and soma in different segments	16%^*2*^
No peripheral, central ascending	11%^*1*^, 16%^*2*^
No peripheral, central descending	62%^*1*^, 42%^*2*^
No peripheral, central ascending and descending	18%^*1*^
Peripheral and central	9%^*1*^

^*1*^GFP^+^ CaP-like cell in *Tg(nrp1a:gfp)js12* E3 morphants. ^*2*^Dye-filled CaP-like cell in *Tg(mnx1:gfp)ml2* E3 morphants. CaP, caudal primary motor neuron; GFP, green fluorescent protein.

### Islet1 knock-down alters sensory neuron properties in the spinal cord and periphery

**Figure 2:**
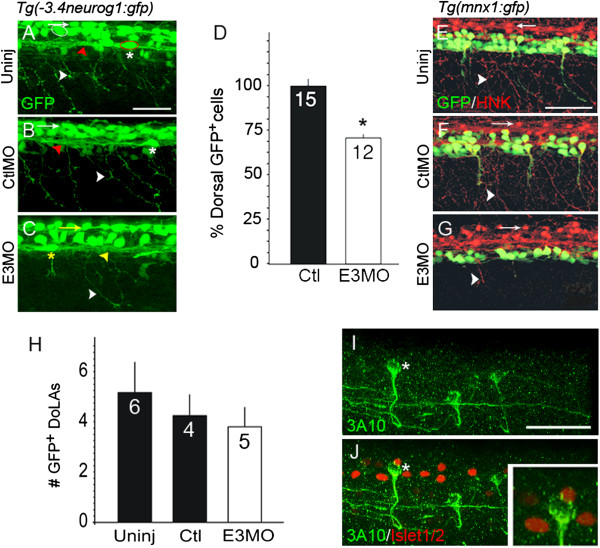
**Islet1 knock-down reduces the number of dorsal GFP**^**+ **^**neurons in the *Tg(-3.4neurog1:gfp)sb4 *line. (A-C)***Tg(-3.4neurog1:gfp)sb4* 24 hours post-fertilization (hpf) Islet1 knock-down reduces the number of dorsal GFP^+^ neurons in the *Tg(-3.4neurog1:gfp)sb4* line embryos express green fluorescent protein (GFP) in Rohon-Beard cells (RBs) [38] (white arrow, white outlined cell as example) and dorsal lateral ascending interneurons (DoLAs; asterisk, red circle). **(A)** In control embryos, central axons (red arrowhead) and peripheral processes (white arrowhead) are GFP^+^. **(B)** The 5-base mismatched *islet1*(Sp)E3 MO (CtlMO) has no effect on RB or DoLA morphology. **(C)** After Islet1 knock-down, central axons (yellow arrowhead) and dorsal cells with RB-like (yellow arrow) or DoLA-like (yellow asterisk) somata remain GFP^+^. However, few peripherally projecting processes are present (white arrowhead in **A** and **B**). **(D)***Tg(-3.4neurog1:gfp)sb4* E3 morphants have 29% less GFP^+^ dorsal neurons versus controls (*P < 0.0001, t-test). GFP^+^ RB neurons were counted in 200 μm spinal cord regions (above yolk sac-yolk sac extension boundary). The number in the bar indicates sample size. **(E-G)***Tg(mnx1:gfp)ml2* embryos were co-processed for GFP and HNK-1-like immunoreactivities to assess both RB (red) and primary motor neuron (PMN; green) peripheral processes. **(E)** RB somata (white arrows), central axons and peripheral processes (white arrowhead) are HNK-1 positive. **(F)** CtlMO has no effect on either motor/interneuron (green) or RB (red) morphology. **(G)** After Islet1 knock-down, few ventral cells express GFP versus uninjected (Uninj) **(E)** or control **(F)** embryos. Few HNK-1^+^ (red) or GFP^+^ (green) projecting peripheral processes are present. **(H)** The number of DoLA interneurons (twelve hemisegments) were counted in 24 hpf control and morphant *Tg(-3.4neurog1:gfp)sb4* embryos. Islet1 knock-down has no effect on the DoLA interneuron number. **(I)** Commissural primary ascending interneurons (CoPAs) are positive for anti-neurofilament antibody 3A10 staining (asterisk). **(J)** In 24 hpf embryos, although several spinal neurons are positive for anti-Islet1/2 immunoreactivity (red), CoPAs are not. Scale bars = 50 μm in **A** (for **A** to **C**), **E** (for **E** to **G**) and **I** (for **I** and **J**).

For morphological study of dorsal spinal neurons, we used the *Tg(-3.4neurog1:*gfp*)sb4* line in which RBs and interneurons express GFP (Figure 2A-C) [38]. Although the identities of all GFP^+^ interneurons in this line are unknown, we find that at least one class has a dorsal lateral ascending axon, the hallmark of the dorsal lateral ascending interneuron (DoLA) [22,37,39]. While both RBs and DoLAs express GFP in this line, they are easily distinguishable from each other on the basis of soma position and axon morphology (Figure 2A-C).

Upon loss of Islet1, dorsal neurons developing in the RB position have few or no peripheral processes [21,26,27] (Figure 2C). We refer to these neurons as RB-like cells to highlight both their similarities (for example, soma size and position) and differences (for example, lack of peripheral processes) with control RB cells. In the *Tg(-3.4neurog1:gfp)sb4* line, Islet1 knock-down leads to ~30% loss of dorsal GFP^+^ neurons with somas in the normal positions of RBs (Figure 2D). GFP^+^ RB and RB-like cells were counted within the 200 μm (~3.5 segments) region of the spinal cord centered about the boundary of the yolk sac and yolk sac extension.

The loss of peripheral processes is reminiscent of the effect of Islet1 knock-down on PMNs (Figure 1). The effects of Islet1 knock-down on both dorsal sensory and ventral motor peripheral axons are simultaneously demonstrated by visualizing RB somas and processes in *Tg(mnx1:gfp)ml2* embryos with the RB marker HNK-1 (Figure 2E-G). Compared to controls (Figure 2E,F), E3 morphants lack processes that innervate the skin as well as peripherally projecting motor axons (Figure 2G).

The number of DoLA interneurons was counted from 12 hemisegments over a region centered on the yolk sac and yolk sac extension. Even though DoLAs express *isl1*[18,19], the number of GFP^+^ DoLAs does not differ significantly between E3 morphants and control *Tg(-3.4neurog1:gfp)sb4* embryos (Figure 2H). Early RNA *in situ* hybridization studies [18,19] raised the possibility that additional interneurons, such as commissural primary ascending interneurons (CoPAs), also express Islet1. However, while we detect Islet1/2 immunoreactivity in several spinal neurons, we do not detect it in CoPAs (Figure 2I,J), suggesting that there may be fewer Islet1 expressing interneurons than initially suggested. The effects of Islet1 knock-down on DoLAs points to important differences between essential roles of Islet1 in inter- versus sensory and motor neurons.

**Figure 3:**
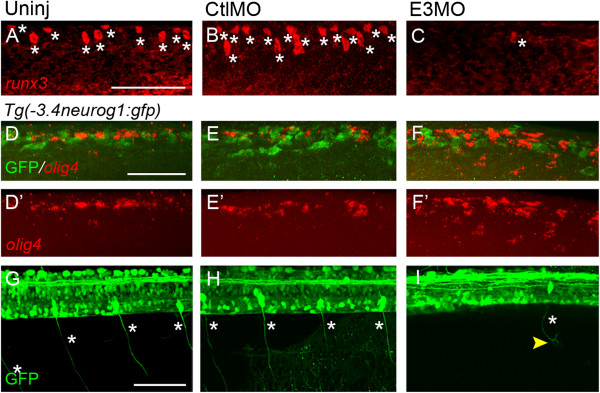
**Islet1 knock-down affects sensory neuron differentiation. (A-C)** In control embryos **(A, B)**, Rohon-Beard cells (RBs) express *runx3*. Islet1 knock-down leads to fewer cells with robust expression of *runx3* (C). The asterisk denotes a cell expressing the gene. **(D-F’)***Tg(-3.4neurog1:gfp)sb4* control **(D-E’)** and E3 morphant **(F, F’)** 24 hours post-fertilization (hpf) embryos were examined for expression of *olig4* (red). **(D-E’)** In lateral views of the dorsal spinal cord, RNA *in situ* hybridization reveals expression of the interneuron marker *olig4* (red). Green fluorescent protein (GFP)^+^ neurons do not express *olig4* and comprise RBs and dorsal lateral ascending interneurons (see Figure 2). **(F-F’)** Islet1 knock-down leads to an increase in the number of *olig4* expressing cells within the dorsal spinal cord. However, similar to RBs of control embryos **(D-E’)**, RB-like neurons do not express detectable levels of *olig4***(F)**. **(G-I)** At 72 hpf, dorsal root ganglia (DRGs) are easily identified as GFP^+^ neurons with large somata located near the spinal cord/notochord border. **(G, H)** In control embryos, DRG neurons project from their soma bipolar axons that extend dorsally and ventrally (asterisks). **(I)** Islet1 knock-down reduces the number of GFP^+^ DRGs. Furthermore, for the few GFP^+^ DRGs remaining, their axons show abnormal morphologies. Scale bars = 50 μm in **A** (for **A-C**), **D** (for **D-F**’) and **G** (for **G-I**). CtlMO, 5-base mismatched; *islet1*(Sp)E3MO; E3MO, E3 morpholino; Uninj, uninjected.

We next tested for effects of Islet1 knock-down on spinal sensory neuron marker expression. *runt* genes play critical roles in sensory neuron differentiation and axon outgrowth (for review, see [40]). *runx3* is expressed within the same level of the spinal cord from which we performed electrophysiological studies [41,42]. Compared to uninjected and control-injected embryos, *runx3* expression is reduced in E3 morphants (Figure 3A-C). Unlike the robust *runx3* expression in RBs of control embryos (8 ± 1 cells, n = 6 embryos, pooled controls), *runx3* transcripts were detected in only a few RB-like cells (0.7 ± 0.7 cells, n = 3 embryos; *P* = 0.003 versus control). These findings indicate that loss of Islet1 reduces expression of a gene essential for primary sensory neuron differentiation.

Another transcription factor, Olig4, represses RB sensory neuron fate, potentially via Islet1 antagonism [43,44]. We tested whether *olig4* expression increases upon loss of Islet1. In both uninjected and control morphant 24 hpf embryos, a population of dorsal interneurons immediately adjacent to RBs normally expresses *olig4* (7.7 ± 1.1 cells, n = 6 embryos; Figure 3D-E’). The dorsal spinal cord region examined had its ventral limit defined by the position of GFP^+^ DoLA interneurons in the *Tg(-3.4neurog1:gfp)sb4 line.* In E3 morphants, however, the number of cells expressing *olig4* is increased (13.7 ± 1.8 cells, n = 3 embryos; *P* = 0.02 versus control; Figure 3D-F’), suggesting an enhanced suppression of sensory neuron fate.

In mice, Islet1 plays a role in differentiation of peripheral sensory neurons, such as dorsal root ganglion (DRG) neurons [17]. To determine if Islet1 is similarly required by zebrafish DRG neurons, we examined effects of Islet1 knock-down on DRG development. At 3 days post-fertilization, control DRG neurons are easily identified in the *Tg(-3.4neurog1:gfp)sb4* by expression of GFP, their bipolar axons and stereotypical soma position lateral to the spinal cord-notochord boundary (Figure 3G,H). Following Islet1 knock-down, less GFP^+^ DRG neurons are present. Furthermore, for GFP^+^ DRG neurons that remain after Islet1 knock-down, their axonal projections are aberrant (Figure 3I). Overall, the results support an essential role for Islet1 in sensory neuron development in zebrafish, in agreement with findings in mammals.

### Islet1 knock-down leads to loss of membrane current properties that characterize ventral motor and dorsal sensory neurons

**Figure 4:**
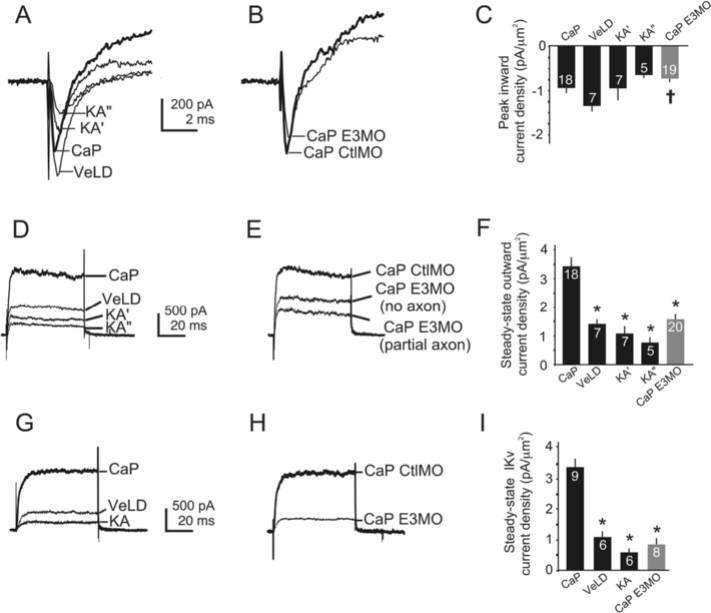
**Caudal primary motor neuron**-**like neurons have voltage-dependent outward current properties that resemble those of ventral lateral descending and Kolmer-Agduhr interneurons rather than caudal primary motor neurons. (A)** In uninjected 24 hours post-fertilization (hpf) embryos, caudal primary motor neurons (CaPs), ventral lateral descending neurons (VeLDs), and Kolmer-Agduhr neurons (KA’s, KA”s) have detectable whole-cell inward current (I_Na/Ca_). **(B)** CaPs of control morpholino (CtlMO) injected and uninjected embryos have I_Na/Ca_ of similar amplitude **(A)**. CaP-like neurons of E3 morphants have I_Na/Ca_ amplitude that does not differ from control CaPs. **(C)** I_Na/Ca_ densities of ventral neurons are not significantly different except for VeLDs versus CaP-like cells (^†^P < 0.05, versus VeLD). **(D)** In control embryos, CaPs have larger amplitude whole-cell outward current (I_Kv/Ca_) than do VeLDs, KA’s or KA”s. **(E)** CaPs of control morphants and uninjected embryos have similar I_Kv/Ca_ density. In contrast, CaP-like neurons, regardless of the presence or absence of a peripheral axon, have I_Kv/Ca_ amplitudes that are smaller than those of control CaPs and resemble those of ventral interneurons **(D)**. **(F)** I_Kv/Ca_ densities are significantly smaller in VeLDs, KA’s and KA”s compared to control CaPs (*P < 0.001 versus CaP). I_Kv/Ca_ of CaP-like versus CaPs is significantly smaller (*P < 0.001 versus CaP), but does not differ from VeLDs, KA’s or KA”s. I_Kv/Ca_ densities do not differ significantly between ventral interneurons. **(G)** In uninjected embryos, CaPs have larger voltage-dependent potassium current (I_Kv_) amplitudes than do ventral interneurons. Outward current properties of KA’ and KA” were not significantly different and are grouped as KA. **(H)** In control morphants (CaP CtlMO injected), CaP I_Kv_ resembles that recorded from CaPs in uninjected embryos **(G)**. In E3 morphants, CaP-like I_Kv_ amplitude is reduced compared to that of control CaPs and more similar to that of ventral interneurons (see **G**). **(I)** CaPs have larger I_Kv_ densities versus interneurons (*P < 0.001 versus CaP). CaP-like neurons have I_Kv_ densities that are significantly reduced versus CaPs (*P < 0.001 versus CaP) but not interneurons.

**Figure 5:**
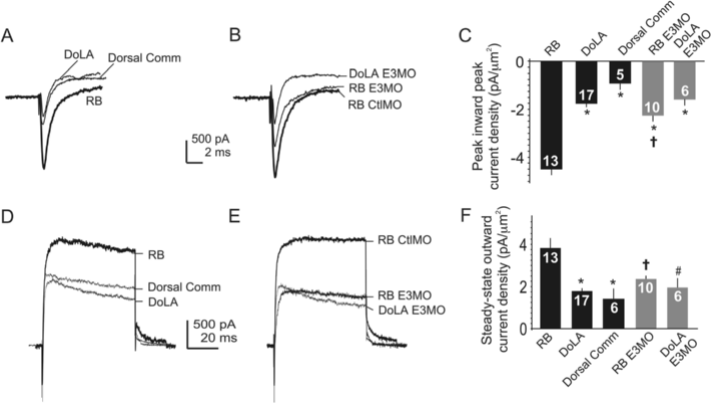
**In E3 morphants, Rohon-Beard-like cells have electrical properties that resemble those of dorsal lateral ascending interneurons and dorsal commissural interneurons. (A)** In uninjected embryos, RB whole-cell inward current (I_Na/Ca_) is of larger amplitude than that of dorsal lateral ascending interneurons (DoLA) or dorsal commissural interneurons (Dorsal Comm). **(B)** In CtlMO injected embryos, RBs have I_Na/Ca_ amplitude that resembles that of RBs in uninjected embryos (see **A**). However, I_Na/Ca_ amplitude of RB-like cells in E3 morphants is smaller than that of control RBs. In contrast, DoLAs in uninjected **(A)** and E3 morphants have similar inward current amplitudes. **(C)** RBs of control embryos have significantly larger inward current density than do dorsal interneurons (*P < 0.001 versus RB). The inward current densities of interneurons in control and E3 morphant embryos are not significantly different from each other. However, I_Na/Ca_ density of RB-like cells is significantly smaller than that of control RBs (*P < 0.001 versus RB) and instead resembles that of DoLAs in control or E3 morphant embryos. In contrast, I_Na/Ca_ density of RB-like cells is significantly larger than that of Dorsal Comm interneurons (^†^P < 0.05 versus Dorsal Comm). **(D)** In uninjected embryos, whole-cell outward current (I_Kv/Ca_) amplitude of RB neurons is larger than that of dorsal interneurons. **(E)** RB-like neurons have smaller amplitude I_Kv/Ca_ than do control RBs in uninjected **(D)** or CtlMO injected embryos **(E)**. In contrast, knock-down of Islet1 has no effect on I_Kv/Ca_ amplitude recorded from DoLAs **(E)**. **(F)** Steady-state I_Kv/Ca_ density of RBs is significantly larger than that of neighboring interneurons (*P < 0.001 versus RB) or of RB-like cells (^†^P < 0.05 versus RB) or DoLAs (^#^P < 0.01 versus RB) in E3 morphants. Current densities of the DoLA and Dorsal Comm interneurons are not significantly different from each other.

The morphological and molecular studies of Figures 1, 2, and 3 suggest that Islet1 promotes differentiation of essential motor and sensory neuron properties. To test this possibility further, we assayed another marker of neuronal identity, electrical membrane properties, as reflected by voltage-dependent currents. We obtained recordings in the whole cell configuration, allowing assessment of membrane currents in the somatic and perisomatic regions. We elicited voltage-dependent currents by briefly bringing the neuron’s membrane potential to values in the range associated with a neuron’s response to inputs and/or firing of action potentials. In the whole-cell voltage-clamp recordings (Figures 4 and 5) we measured the peak amplitude of the inward current, I_Na/Ca_, and amplitude of the outward current, I_Kv/Ca_, elicited at +40 mV, a value achieved during the peak of an action potential. In Figures 4 and 5 we present examples of currents that were elicited from individual neurons. In addition, the data are summarized in bar graph form showing current densities, a value that normalizes current amplitude to cell surface area (see Methods).

For study of ventral neurons, we recorded from CaP and three ventral interneurons types, VeLD, KA’ and KA”, because the axonal tracks of CaP-like cells are often similar to those of VeLDs, KA’s and KA”s (Figure 1). Moreover, previous work indicates that single ventral spinal cord precursor cells can give rise to a PMN as well as a VeLD or KA’, demonstrating a shared lineage for two of these ventral interneurons [45-48]. Furthermore, Islet1 knock-down leads to the appearance of a novel population of ventral neurons with somas in PMN-like positions but positive for markers of GABAergic neurons, such VeLDs, KA’s and KA”s [13,49].

We used the *Tg(mnx1:gfp)ml2* line to record from CaP and VeLD in control embryos and CaP-like cells in E3 morphants (Figure 1; Additional file 1). We included AlexaFluor 594 in the pipette solution to allow for dye filling of the recorded neuron and visualization of its morphology as an additional test of cell identification (for example, Figure 1C; see Methods). Nonetheless, it is possible that the properties that we used to identify VeLD might have led to inclusion of ICs [23,30]. However, none of the presumed VeLDs from which we recorded displayed the distinctive firing properties of IC neurons [50]. This may reflect that our recordings were obtained from segments above the yolk sac rather than more rostral ones where spinal ICs reside [23,30]. On this basis, we refer to the identified interneurons with descending axons in *Tg(mnx1:gfp)ml2* embryos as VeLDs.

Although KA’ and KA” neurons have similar names, they reside in different regions of the ventral spinal cord, with KA’s more dorsal than KA”s [47]. Both KA’ and KA” neurons are readily identifiable on the basis of position and GFP expression in the *Tg(8.1kGata1:eGFP)* line (Additional file 1) [48]. We present data for both the motor neuron lineage-related KA’ group as well as KA” neurons.

In control embryos, the amplitudes and densities of I_Na/Ca_ do not differ significantly between CaPs and the interneurons (Figure 4A-C). With respect to outward current, CaPs have greater I_Kv/Ca_ amplitude and density than do VeLD, KA’ or KA” interneurons (Figure 4D-F). I_Kv/Ca_ comprises a voltage-dependent component, I_Kv_, as well as a calcium- and voltage-dependent one, I_KCa_. In order to assess which component of I_Kv/Ca_ differs between these neurons, we also performed recordings in the presence of blockers to isolate the I_Kv_ component (Figure 4G-I; see Methods). These results indicate that the greater amplitude of CaP versus VeLD, KA’ or KA” I_Kv/Ca_ largely reflects a larger voltage-dependent component, I_Kv_, of the outward current (Figure 4G-I).

We next measured current amplitudes of CaP-like cells in E3 morphant embryos. Following Islet1 knock-down, CaP-like neurons have I_Na/Ca_ amplitudes and densities that are similar to those of CaPs, KA’s and KA”s (Figure 4A-C). Further, CaP-like neurons have a reduced I_Na/Ca_ density versus that of VeLDs.

With respect to I_Kv/Ca_, its amplitude and density in CaP-like cells differs from that of CaPs (Figure 4D-F). These differences occur regardless of whether CaP-like neurons had extended a peripheral axon by the time of recording (Figure 4E). In contrast, CaP-like outward current properties do not differ from those of VeLDs, KA’s or KA”s. Further, the differences in outward current properties between CaPs, VeLDs, KA’s, KA”s and CaP-like cells are largely due to the voltage-dependent component, I_Kv_ (Figure 4G-I).

In the dorsal spinal cord, recent work has shown that the domain organization of the dorsal spinal cord in zebrafish is conserved with other vertebrates [51]. Nonetheless, the choice of interneurons from which to record was less obvious than in the ventral cord, because lineage relationships among RBs and neighboring interneurons are poorly understood. Accordingly, we limited our consideration to primary interneurons (for example, DoLAs and CoPAs) [22,37,39,52].

As mentioned, both RBs and DoLAs express *isl1*[18,19]. Furthermore, in the *Tg(-3.4neurog1:gfp)sb4* line, both RBs and DoLAs express GFP (Figure 2). On this basis, DoLA was one dorsal interneuron type that we chose to study. However, in contrast to glutamatergic RBs, DoLAs are GABAergic. In view of this difference, we also recorded from a second dorsal interneuron group that we refer to here as dorsal commissural interneurons (Dorsal Comms). We identified Dorsal Comms via dye filling as dorsal interneurons with commissural axons that project ventrally from the soma towards the midline. In several cases, the dye fill was extensive enough to allow visualization of the axon after the midline was crossed. In these cells, the Dorsal Comm axon projected rostrally after crossing the midline, revealing the morphological signature of the commissural primary ascending interneuron, CoPA [22,37,52]. Thus, the Dorsal Comm group includes (1) glutamatergic CoPAs [49], (2) cells that are potentially CoPAs but not sufficiently dye filled to allow detection of the ascending portion of the axon on the contralateral side, and (3) possibly a few later born secondary interneurons (known as commissural secondary ascending interneurons; CoSAs) potentially present at 24 hpf. To avoid inclusion of CoSAs in the Dorsal Comm group, we took advantage of the larger soma size of primary (for example, CoPA) versus secondary (for example, CoSA) neurons. All unambiguously identified CoPAs had cell capacitance greater than 5 pF, the minimum value that we set as an inclusion criterion for the Dorsal Comm group.

For 24 hpf control embryos, the properties of voltage-dependent currents differ between RBs and the interneurons (Figure 5A-F). In comparison to RBs, both Dorsal Comms and DoLAs have significantly smaller I_Na/Ca_ amplitudes and densities (Figure 5A-C). With respect to outward currents, RBs have significantly larger I_Kv/Ca_ amplitudes and densities than do Dorsal Comms or DoLAs (Figure 5D-F). Thus, RBs have substantially larger inward and outward current densities than do neighboring interneurons.In 24 hpf E3 morphants, we recorded from dorsal RB-like cells. Compared to RBs, RB-like cells have significantly smaller inward and outward current amplitude and density (Figure 5), a profile that is more similar to those of DoLAs and Dorsal Comms rather than RBs. This is similar to the situation in the ventral cord, where CaP and CaP-like cells have clearly different excitable membrane properties.

In 24 hpf E3 morphants, we recorded not only from RB-like cells but also DoLAs because both of these dorsal neurons express *isl1*. Islet1 knock-down has no detectable effect on inward or outward current properties of DoLAs (Figure 5). Similarly, loss of Islet1 does not affect the number of DoLAs in E3 morphants (Figure 2). These results suggest that the role of Islet1 in DoLAs can be compensated by another factor or that it has a substantially different role in interneurons compared to RBs (or CaPs).

Overall, under conditions of reduced Islet1 expression, the voltage-dependent current properties of CaP-like and RB-like cells differ from those of CaPs and RBs. These findings are consistent with a role for Islet1 in development of both sensory and motor neuron electrical membrane properties. In many respects, CaP-like and RB-like neurons have voltage-dependent properties that resemble those of neighboring interneurons rather than CaPs or RBs, respectively.

### Molecular markers of channel and neurotransmitter gene expression

A major difference between RB and RB-like current properties is the amplitude and density of the rapidly activating inward current. Although I_Na/Ca_ reflects both inward voltage-gated sodium and calcium currents, by measuring the peak amplitude we essentially assess voltage-gated sodium current (see Methods). In RBs, the Nav1.6a sodium channel (encoded by the *scn8aa* gene) accounts for the majority of sodium current [53,54]. We examined whether Islet1 knock-down affects expression of *scn8aa*.

**Figure 6:**
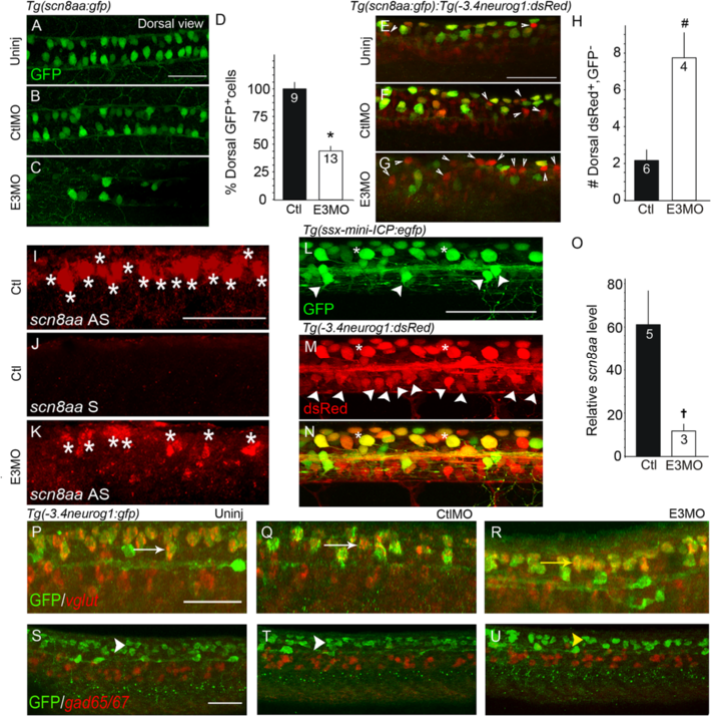
**Islet1 knock-down reduces Rohon-Beard cell number and expression of *scn8aa *in the dorsal spinal cord. (A-C)** In 24 hours post-fertilization (hpf) *Tg(scn8aa:gfp)ym1 embryos*, Rohon-Beard (RB) and RB-like cells express green fluorescent protein (GFP) *(dorsal views)*[46]. Islet1 knock-down reduces the number of GFP^+^ neurons (**C** versus **A** and **B**). **(D)** In E3 morphants, the number of GFP^+^ RB-like cells is 44% that of GFP^+^ RB cells in controls (*P < 0.001) (see Methods). **(E-G)** In uninjected (Uninj) and control 24 hpf double *Tg(scn8aa:gfp,-3.4neurog1:dsRed)* embryos, most RB neurons express both reporters **(E, F)** and few express only dsRed (arrowheads) (lateral views). In E3 morphants, many RB-like cells express only dsRed (**G**, arrowheads). **(H)** Compared to RBs, more RB-like cells express only dsRed (^#^P < 0.01). **(I-K)** Following Islet1 knock-down **(K)**, less *scn8aa* mRNA (asterisks) is detected compared to controls **(I)** (lateral views). **(J)***The* sense control *scn8aa* probe reveals little signal, indicating specificity of the antisense probe (**I** and **K**). **(L-N)** In 24 hpf double transgenic *Tg(ssx-mini-ICP:egfp,-3.4neurog1:dsRed)* embryos, most RB cells (asterisks) express both reporter proteins **(N)**. Fewer ventral interneurons express GFP **(L)** versus dsRed **(M)** (arrowheads). **(O)** Quantitative reverse transcription PCR analysis (see Methods) shows a five-fold reduction in *scn8aa* expression in RB-like cells of E3 morphants (*P < 0.04). **(P**, **Q)** RBs express *vglut2.1/2.2* mRNA (red) (lateral views). In uninjected and CtlMO injected embryos, *vglut2.1/2.2* mRNA (red) colocalizes (white arrows) with GFP^+^ RBs (green). **(R)** In E3 morphants, *vglut2.1/2.2* mRNA (red) co-localizes with GFP^+^ RB-like neurons (green; yellow arrow), indicating no obvious effect of Islet1 knock-down. **(S**, **T)** GABAergic neurons express *gad65/67* mRNA (red). **(U)** Islet1 knock-down has no obvious effect on *gad65/67* in the dorsal domain. Scale bars = 50 μm in **A** (for **A** to **C**), **E** (for **E** to **G**), **I** (for **I** to **K**), **L** (for **L** to **N**), **P** (for **P** to **R**) and **S** for (**S** to **U**).

As a first approach to investigate this possibility, we took advantage of the *Tg(scn8aa:gfp)ym1* line [55], in which the *scn8aa* promoter drives expression of GFP in RBs (Figure 6A,B). We first compared the number of GFP^+^ RBs present in control *Tg(scn8aa:gfp)ym1* and *Tg(-3.4neurog1:gfp)sb4* 24 hpf embryos to determine whether similar RB populations are identified (Figures 2 and 6). The numbers of GFP^+^ RB neurons are similar (30 ± 2 and 30 ± 1, respectively), suggesting that the two promoters drive expression of GFP in a similar number of RBs.

After knock-down of Islet1, both transgenic lines have less GFP^+^ RB-like cells versus the number of RBs found in controls (Figures 2A-D and 6A-D). This result is consistent with our finding of reduced *runx3* expression in E3 morphants (Figure 3). However, the numbers of GFP^+^ RB-like cells in the two transgenic morphants differ, with E3 *Tg(scn8aa:gfp)ym1* morphants having significantly less (Figure 6A-D versus Figure 2A-D; *P* < 0.001). This finding suggests that loss of Islet1 not only affects the number of sensory neurons that differentiate but also *scn8aa* expression. To assess more directly whether RB-like cells persist in E3 morphants despite reduced GFP expression in the *Tg(scn8aa:gfp)ym1* line, we crossed this line into the *Tg(-3.4neurog1:dsRed)* line. In control embryos, there are few RBs that are dsRed^+^ and GFP^-^ (Figure 6E,F, arrowheads). However, after Islet1 knock-down, many RB-like cells are GFP^-^ despite being dsRed^+^ (Figure 6G arrowheads and H), suggesting less activation of the *scn8aa* promoter upon knock-down of Islet1. RB counts were performed as previously described over segments encompassing the yolk sac and yolk sac extension.

We further tested the possibility that Islet1 knock-down affects *scn8aa* expression via RNA *in situ* hybridization. Islet1 knock-down leads to a clear reduction in the *scn8aa* RNA signal within the dorsal domain of E3 morphants compared to controls (Figure 6I-K). To analyze *scn8aa* expression quantitatively, we performed quantitative reverse transcription PCR analysis for *scn8aa* using RNA extracted from fluorescence-activated cell (FAC) sorted RB cells of control and E3 morphant embryos (see Methods). For these studies, we used the *Tg(ssx-mini-ICP:egfp)* line that has GFP expression predominantly in RBs with few GFP^+^ interneurons (Figure 6L-N). The relative levels of *scn8aa* transcripts (normalized to *eef1α1a*) were five-fold greater in control versus E3 morphant FAC sorted GFP^+^ cells (Figure 6O; *P* < 0.04). Thus, not only are there less RB-like cells than RBs in E3 morphant versus control embryos, but expression of *scn8aa* is also reduced in RB-like cells compared to RBs.

In the ventral spinal cord, Islet1 knock-down alters another property of neuronal signaling, neurotransmitter expression [13]. We tested whether Islet1 affects expression of *vglut* and *gad65/67,* genes that regulate the levels of the excitatory transmitter, glutamate, and the inhibitory transmitter, γ-aminobutyric acid (GABA), respectively. However, the expression patterns of *vglut* and *gad65/67* in the dorsal spinal cord of E3 morphants do not show any obvious changes (Figure 6P-U).

In summary, in the dorsal spinal cord, Islet1 knock-down alters expression of a critical voltage-gated ion channel gene, *scn8aa*, and the density of I_Na/Ca_, but does not produce a notable change in the expression of genes that determine neurotransmitter phenotype.

### CaP-like and RB-like neurons fire action potentials with novel properties

In both the ventral and dorsal cord, interneurons differ from CaPs and RBs with respect to the properties of their voltage-dependent currents (Figures 4 and 5). These differences predict that interneurons will fire action potentials that differ from those of CaPs and RBs.

**Figure 7:**
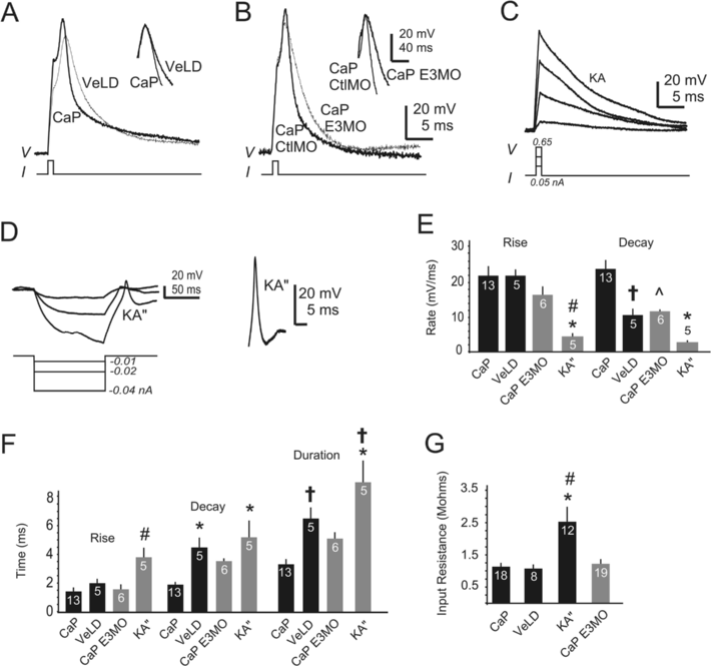
**Caudal primary motor neuron and caudal primary motor neuron**-**like neurons fire action potentials with different properties. (A, B)** Action potentials were evoked from caudal primary motor (CaPs), ventral lateral descending (VeLDs) and CaP-like neurons (see Methods). The insets align action potentials at their peaks to highlight kinetic differences. **(A)** During an action potential, the membrane potential repolarizes faster in CaPs than it does in VeLDs. **(B)** For CaP action potentials, the 5-base mismatched *islet1*(Sp) E3 morpholino (CtlMO) has no obvious effect on repolarization. In comparison, CaP-like action potentials of E3 morphants repolarize slowly. **(C)** Kolmer-Agduhr neurons (KAs) do not fire action potentials in response to brief depolarizing current injections. **(D)** After prolonged injection of hyperpolarizing current, KA”s fire regenerative responses. A single regenerative response recorded from another KA” is enlarged (right). **(E)** KA” action potentials have slower rates of rise than those elicited from CaPs (*P < 0.001 versus CaP) or VeLDs (^#^P < 0.01 versus VeLD). However, the rate of rise of CaP-like action potentials is not significantly different from that of KA”s, VeLDs or CaPs. In contrast, the rate of decay of CaP action potentials is significantly faster than that of VeLD (^†^P < 0.01 versus CaP), KA”s (*P < 0.001 versus CaP) or CaP-like action potentials (^P < 0.01 versus CaP). **(F)** Regenerative responses fired by KA”s have prolonged rise (^#^P < 0.01 versus CaP or CaP E3MO) and decay (*P < 0.001 versus CaP) times. VeLDs fire action potentials of significantly longer duration than do CaPs (^†^P < 0.05 versus CaP). Similarly, KA” regenerative responses have significantly longer durations than do those elicited from CaPs (*P < 0.001 versus CaP) or CaP-like cells (^†^P < 0.05 versus CaP E3MO). **(G)** CaPs, VeLDs and CaP-like neurons have similar input resistance. Compared to CaP, KA”s have significantly greater input resistance (*P < 0.001 versus CaP) or CaP-like cells (^#^P < 0.01 versus CaP E3MO).

We first examined this prediction in the ventral spinal cord. In control embryos, the most notable differences between CaP and VeLD, KA’ or KA” currents are the larger densities of I_Kv_/_KCa_ and I_Kv_ recorded from CaPs (Figure 4D-I). These differences predict faster repolarization and shorter durations for impulses elicited from CaPs versus the ventral interneurons. Consistent with this prediction, CaP action potentials show faster rates of repolarization than do those of VeLDs (Figure 7A,E).

**Figure 8:**
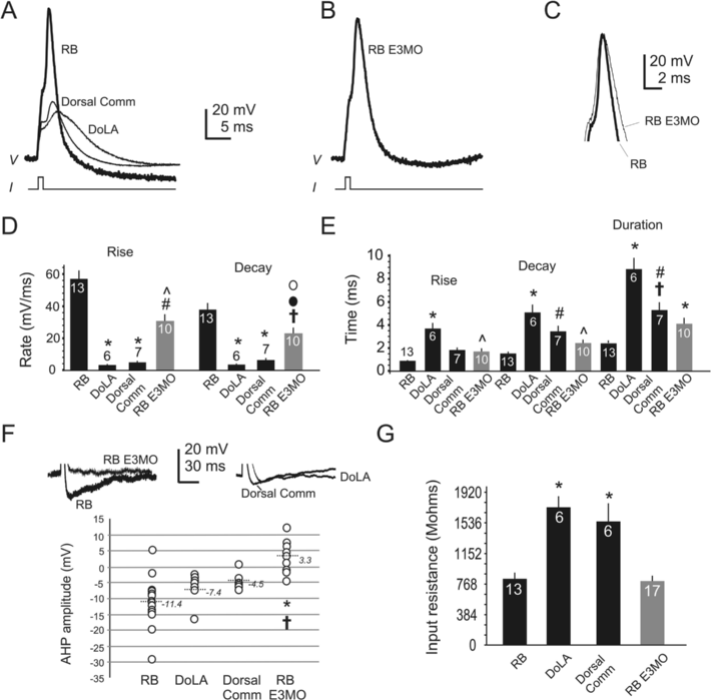
**Rohon-Beard and Rohon-Beard-like cells fire action potentials with different properties. (A, B)** Action potentials were evoked from dorsal spinal neurons in 24 hours post-fertilization (hpf) embryos (see Methods). **(A)** Rohon-Beard (RB) action potentials waveform have a distinctive overshoot and afterhyperpolarization (AHP). **(B)** RB-like cells of E3 morphants fire action potentials without a prominent AHP. **(C)** Aligning the peaks of RB and RB-like action potentials highlights kinetic differences. **(D, E)** Several properties of action potentials (rate of rise, rise time, rate of decay, decay time, duration) were evaluated at rheobase. **(D)** RBs fire action potentials with faster rates of rise and decay than those elicited from dorsal lateral ascending interneurons (DoLAs) and dorsal commissural interneurons (Dorsal Comms) (*P < 0.001 versus RB). Compared to RBs, RB-like cells fire action potentials with slower rates of rise (^#^P < 0.01 versus RB) and decay (^†^P < 0.05). Compared to DoLAs and Dorsal Comms, RB-like cells fire action potentials with faster rates of rise (^P < 0.01 versus DoLAs and Dorsal Comms) and decay (^•^P < 0.01 versus DoLA; °P < 0.05 versus Dorsal Comm). **(E)** Compared to RBs, DoLAs fire action potentials with increased rise time, prolonged decay time and longer duration (*P < 0.001 versus RBs). Dorsal Comms fire action potentials with longer decay time (^#^P < 0.01) and duration (^†^P < 0.05) than do RBs. DoLAs and Dorsal Comms fire impulses that have significantly different durations (^#^P < 0.01 versus DoLA). RB-like cells fire impulses with decreased rise times and briefer decay time than do DoLAs (^P < 0.001 versus DoLAs). **(F)** RB-like cells fire impulses with small AHP amplitudes in contrast to those of RBs (*P < 0.001) or DoLAs (^†^P < 0.05). **(G)** The input resistances of RB-like and RB cells are not significantly different but significantly lower than those of DoLAs and Dorsal Comms (*P < 0.001).

For both CaPs and VeLDs, action potentials are elicited by injections of brief depolarizing current (Figure 7A,B). In contrast, KA”s do not fire action potentials in response to depolarizing current injections (Figure 7C). Instead, injection of prolonged hyperpolarizing current is required to trigger a regenerative response from KA”s (Figure 7D)*.* The rate of depolarization and repolarization for the KA” regenerative response is significantly slower than that of the CaP action potential, resulting in a longer duration (Figure 7E,F). CaPs and VeLDs also differ from KA”s with respect to input resistance, with KA”s having a significantly larger value (Figure 7G). We did not record action potentials from KA’ interneurons.In E3 morphants, injection of brief depolarizing current is sufficient to elicit action potentials from CaP-like neurons (Figure 7B). In this regard, CaP-like cells resemble CaPs and VeLDs but not KA”s. However, CaP-like cells have voltage-dependent outward current properties that resemble more those of VeLDs than CaPs (Figure 4), predicting that CaP-like action potentials might have durations more similar to those of VeLD rather than CaPs. Indeed, CaP-like action potentials repolarize significantly more slowly than do those of CaPs but not differently from those of VeLDs (Figure 7A,B,E,F).We next compared action potentials fired by the dorsal neurons: RBs, DoLAs, Dorsal Comms and RB-like cells (Figure 8). RBs fire action potentials with large amplitude overshoots (Figure 8A). In contrast, DoLA and Dorsal Comm action potentials do not overshoot (Figure 8A). The rates of rise and decay of RB action potentials are faster than those of DoLAs and Dorsal Comm interneurons (Figure 8D), resulting in briefer action potential durations for RBs versus DoLAs and Dorsal Comms (Figure 8E). A prominent afterhyperpolarization (AHP) further distinguishes RB action potentials from those of the interneurons (Figure 8A-F).In E3 morphants, the rates of rise and decay of RB-like action potentials are slower compared to those of RBs (Figure 8B-D). Further, in contrast to RBs, RB-like cells fire action potentials that rarely have AHPs (Figure 8B-F). When AHPs are present, they are of smaller amplitude compared to those of RB action potentials (Figure 8F). Further, the input resistance of RB-like neurons differs significantly from that of DoLAs and Dorsal Comms, but not from that of RBs (Figure 8G). These data indicate that RB-like cells not only have altered morphology that would prevent normal function as a primary sensory neuron, but also non-RB-like electrophysiological properties.

Overall, on the basis of morphological, molecular and electrophysiological properties, CaP-like and RB-like cells have properties that distinguish them from control CaPs and RBs, respectively. These data support a role for Islet1 in determining multiple neuronal properties that define neuronal identity.

## Discussion

Our studies of the effects of Islet1 on development of zebrafish spinal neurons using electrophysiological methods provide two major findings. First, electrical membrane properties uniquely identify several neuronal subtypes that develop in the embryonic zebrafish spinal cord. In the ventral cord, CaP distinguishes itself from neighboring interneurons by its large outward current density (Figure 4) and firing action potentials with briefer decay times (Figure 7). In the dorsal domain, interneurons have smaller inward and outward conductances than do RBs (Figure 5). Similarly, in the embryonic *Xenopus* spinal cord, RBs have larger outward conductances than do dorsal interneurons [56]. Further, the action potentials fired by RBs, DoLAs and Dorsal Comms differ (Figure 8), consistent with their characteristic inward and outward conductances. RBs fire action potentials that repolarize rapidly and are followed by prominent AHPs. In contrast, DoLAs and Dorsal Comms fire non-overshooting impulses that repolarize slowly and have small amplitude or no AHPs. Thus, these basic electrical membrane properties distinguish spinal neurons from each other.The second major finding concerns the role of Islet1 in neuronal differentiation, and the insights provided by assaying effects on electrical membrane properties. CaP-like cells of E3 morphants differ from control CaPs by having significantly reduced outward current density, the property that distinguishes CaP from neighboring interneurons (Figure 4). Similarly, in the dorsal cord, RB-like cells differ from RBs by having reduced inward and outward current densities (Figure 5), properties that distinguish RBs from neighboring interneurons. On this basis, Islet1 is required for differentiation of electrical membrane signatures that identify CaPs as well as RBs.

We studied three neuronal subtypes in the spinal cord that express *isl1*: CaP, RB and DoLA. Even though all express *isl1*, their electrical membrane properties differ. While CaP and RB have similar outward current densities, they differ with respect to inward current density (Figures 4 and 5). DoLA differs from both CaP and RB by its much smaller densities of inward and outward currents (Figures 4 and 5) and inability to fire overshooting action potentials (Figure 8). Moreover, Islet1 does not appear to be required for differentiation of electrical membrane properties of DoLA, as electrical membrane properties of this interneuron do not differ between control and E3 morphants (Figure 5). How might this occur? One possibility is that in CaPs and RBs, the binding partners of Islet1 allow it to have an essential function in complexes that directly or indirectly lead to activation of ion channel gene expression that is required for their subtype-specific excitability. In contrast, in DoLAs, loss of Islet1 may be compensated by another factor or Islet1 may interact with different partners and function in complexes that have roles unrelated to specification of electrical membrane properties. Overall, these considerations emphasize the importance of cellular context in the contributions that Islet1 makes to differentiation programs [11,12,16,57-60].

For vertebrate motor neuron specification, Islet1 participates in a hexamer complex together with LIM homeobox 3 (Lhx3) and nuclear LIM interactor (NLI) protein [57-59]. Furthermore, a recent study shows that other transcription factors, such as STAT3, may interact with the Islet1/Lhx3/NLI hexamer complex and also collaborate in motor neuron differentiation [61]. Interestingly, in zebrafish, CaPs express activated phosphorylated STAT3 (pSTAT3), and loss of pSTAT3 function leads to defects in axonal pathfinding. However, there are no defects in motor neuron specification, suggesting that STAT3 itself, or in a complex, regulates aspects of differentiation that occur later than motor neuron genesis [62].

In vertebrates, little is known about the direct targets of Islet1 and how they ultimately lead to regulation of ion channel gene expression. In *Drosophila*, however, expression of the Islet1 orthologue, Islet, in motor neurons leads to a decrease in the density of an inactivating potassium current [8]. The *Shaker* gene encodes an inactivating potassium channel, and Islet binds directly to the *Shaker* locus. Further, another HD transcription factor, Lim3, also binds to the *Shaker* locus, suggesting that these two transcription factors act together to repress transcription of the Shaker gene [63]. Interestingly, Islet functions to repress *Shaker* expression and reduce potassium current density in *Drosophila* motor neurons, whereas we find that loss of Islet1 leads to decreased outward current density. While the direct targets of Islet1 in zebrafish are not known, it is clear that Islet1 is required for generation of the normal large potassium current density that characterizes both CaPs and RBs. These considerations suggest that Islet1 may act to increase potassium channel gene expression in zebrafish.

Prior studies have demonstrated a requirement for Islet1 for proper expression of ion channel genes in vertebrate sensory neurons. In sensory neurons of Islet1 knock-out E12.5 mice, there is reduced expression of *SCN10A*, a sodium channel expressed predominantly in DRG neurons [17]. Using conditional knock-out methods to limit Islet1 excision to stages after E11.5, subsequent microarray analyses reveal reduced expression in DRG neurons of several ion channel genes including *SCN7A, SCN9A, TRPV1*[64]. Similarly, we find reduced expression of *scn8aa* (orthologous to mammalian *SCN8A*) in sensory RB cells after knock-down of Islet1 (Figure 6). Thus, several studies implicate Islet1 in regulation of voltage-gated sodium channel expression. Future studies will identify downstream targets of Islet1 and whether Islet1 directly or indirectly regulates transcription of *scn8aa*.

## Conclusions

Overall, our findings support the view that electrical membrane properties provide another set of markers to identify zebrafish spinal neurons. Furthermore, these electrical membrane markers are impacted upon loss of Islet1. The specific membrane properties that are affected by Islet1 knock-down differ amongst the neurons studied, presumably reflecting the combinatorial nature of HD transcriptional regulation of genetic programs.

## Methods

### Animal care and zebrafish transgenic lines

Adult zebrafish (*Danio rerio*) were maintained at 28.5°C on a 14 hour light/10 hour dark cycle in the Center for Comparative Medicine at the University of Colorado Anschutz Medical Campus and bred using standard protocols [65]. The University of Colorado Committee on Use and Care of Animals approved all animal protocols. Embryos were kept in embryo media (in mM: 130 NaCl, 0.5 KCl, 0.02 Na_2_HPO_4_, 0.04 KH_2_PO_4_, 1.3 CaCl_2_, 1.0 MgSO_4_, 0.4 NaH_2_CO_3_) and staged according to external morphology [66].

### Transgenic lines

Various transgenic lines were used to facilitate identification of neurons in morphological and gene expression studies as well as for electrophysiological recordings. For recordings from dorsal neurons, the *Tg(-3.4neurog1:gfp)sb4* line [38] was used (kindly provided by Dr Uwe Strähle, University of Heidelberg, Heidelberg, Germany). Wild-type embryos were also used for RB recordings because these cells are easily recognized on the basis of their large soma and dorsal position within the spinal cord [67]. For recordings from ventral neurons, the *Tg(mnx1:gfp)ml2*[35] (Zebrafish International Resource Center, Eugene, OR, USA) and *Tg(8.1kGata1:eGFP)* (generously provided by Dr Katherine Lewis, Syracuse University, Syracuse, NY, USA) lines were used [34,48,68]. *Tg(ssx-mini-ICP:eGFP)*[69], *Tg(nrp1a:gfp)js12*[35] and *Tg(scn8aa:gfp)ym1*[55] lines were also used for immunohistochemical, RNA *in situ* hybridization and FAC sorting analyses and kindly provided by Drs Hiroshi Okamoto (RIKEN, Saitama, Japan), Waturu Shoji (Tohoku University, Sendai, Japan) and the Zebrafish International Resource Center, respectively.

### Morpholinos

Knock-down of Islet1 expression was achieved by use of the previously reported *islet1*(Sp)E3MO (E3MO), which binds to the junction between exon3/intron3 ('5-GAATGCAATGCCTACCTGCCATTTG-3') [13]. To control for possible off-target effects, a control 5-mispaired morpholino (CtlMO) was designed to the same exon3/intron3 junction ('5-GAATcCAATcCCTAgCTGCgATaTG-3') (GeneTools, LLC, Philomath, OR, USA). Embryos injected with E3MO or CtlMO are referred to as E3 or Ctl morphants, respectively. The efficacy of the E3MO injections was assayed by Islet1/2 immunoreactivity and *islet1* mRNA *in situ* hybridization (Additional file 2), as performed previously [13]. Embryos were grouped on the basis of the severity of the motor neuron axonal phenotype, which was assessed by the use of the *Tg(mnx1:gfp)ml2* line.

### Immunohistochemistry and confocal microscopy

Whole mount immunohistochemistry was performed as described previously [70-72]. Briefly, when embryos reached the desired developmental stage, they were fixed in 4% paraformaldehyde. Fixed preparations were permeabilized by incubation in water, followed by treatments with acetone, and then collagenase (1 mg/mL). Incubation with appropriate antibodies (Table 2) was overnight at 4°C. Specimens were mounted laterally with fluoromount (SouthernBiotech, Birmingham, AL, USA) and imaged with a LSM5 Pascal Confocal Microscope (Carl Zeiss, Inc., Thornwood, NY, USA) equipped with a 40 or 63× water immersion lens (0.8 and 0.95 numerical aperture, respectively). Confocal z-stacks comprising 1 μm sections were obtained and collapsed to create projections of one side of the spinal cord, using LSM software (Carl Zeiss, Inc.) or Image J [73] unless otherwise indicated.

**Table 2:** **RNA *in situ *** **hybridization and antibody probes**

**RNA/antibody**	**Properties**	**Source**
*islet1*	1,900 bp	Dr Judith Eisen, Institute of Neuroscience, University of Oregon, Eugene, OR, USA
*nrp1a*	1,200 bp	Dr Waturu Shoji, Department of Cell Biology, Institute of Development, Aging and Cancer, Tohoku University, Sendai, Japan
*vglut2.1*	500 bp	Dr Shin-ichi Higashijima, Okazaki Institute of Integrative Bioscience, Okazaki, Japan
*vglut2.2*	500 bp	
*gad65*	900 bp	
*gad67*	900 bp	
*olig4*	1000 bp	Dr Francesco Argenton, Department of Biology, University of Padua, Padua, Italy
*scn8aa*	655 bp	Our work
*runx3*	1,900 bp	Dr Phil Crosier, Dept Mol Medicine, University of Auckland, Auckland, New Zealand
anti-GFP	Rabbit polyclonal, (1:700)	Molecular Probes-Life Technologies, Grand Island, NY, USA
anti-zn12 (HNK)	Mouse monoclonal, (1:100)	DSHB*
anti-GABA	Rabbit polyclonal, (1:250)	Sigma-Aldrich Co., St. Louis, MO, USA
goat anti-rabbit	Conjugated to Alexa-568/488, (1:1000)	Molecular Probes-Life Technologies
goat anti-mouse	Conjugated to Alexa-488, (1:1000)	Molecular Probes-Life Technologies

*DSHB, Developmental Studies Hybridoma Bank; the zn12 monoclonal antibody was developed under the auspices of the National Institute of Child Health and Development and maintained by The University of Iowa, Department of Biological Sciences, Iowa City, IA, USA. bp, Base pair; GABA, γ-aminobutyric acid; GFP, green fluorescent protein.

### RNA *in situ* hybridization

RNA *in situ* hybridization was carried out on whole mount 24 hpf embryos using standard methods and digoxigenin-labeled probes [70,74,75]. The genes studied are listed in Table 2. To identify glutamatergic or GABAergic neurons, we followed the method of Higashijima and colleagues [49] involving simultaneous use of probes for either two different vesicular glutamate transporters, *vglut2.1* and *vglut2.2,* or two forms of glutamic acid decarboxylase, *gad65* and *gad67*. Probe hybridization was detected by use of anti-digoxigenin-AP Fab fragments (Roche, Indianapolis, IN, USA) in combination with the chromogen Fast Red (Sigma, St. Louis, MO, USA) [75]. Embryos were mounted laterally for confocal imaging. To count the number of cells expressing *runx3* or *olig4*, images were imported into Image J [73] and expressing cells were identified by finding maxima.

### Fluorescence-activated cell sorting of RB cells

For dissociation of cells in preparation for FAC sorting, we followed a published protocol [76]. We used the *Tg(ssx-mini-ICP:egfp)* line, because predominantly RBs and only few interneurons within the spinal cord express GFP [69]. Briefly, 24 to 26 hpf embryos were immobilized with 0.01% tricaine (ethyl 3-aminobenzoate methanesulfonate salt, Sigma-Aldrich, St Louis, MO, USA) and mounted in 0.5% low melting agarose, UltraPure™ LMP Agarose (Gibco BRL, Life Technologies Corporation, Carlsbad, CA, USA). Tungsten needles were used to severe the heads (to remove GFP^+^ trigeminal neurons) and trunks were collected from the agarose by rinsing with 0.5× Danieau’s solution (in mM: 29 NaCl, 0.35 KCl, 0.2 MgSO_4_.7H_2_O, 0.3 Ca(NO_3_)_2_, 2.5 HEPES). Following several rinse-centrifugation steps with 0.5× Danieau’s solution, trunks were dissociated by incubation in 1× Thermo Scientific™ HyClone™ Trypsin-EDTA (Thermo Fisher Scientific, Wilmington, DE, USA). Dissociated tissue was centrifuged and resuspended in FACSmax cell dissociation solution (AMS Biotechnology, Abingdon, UK) and strained through a 40 μm Falcon™ cell strainer (Thermo Fisher Scientific) to produce the final cell suspension. The UCAMC Flow Cytometry Core, part of the Gates Regenerative Medicine and Stem Cell Biology Center, performed FAC sorting using a MoFlo XDP (Beckman Coulter) cell sorter equipped with a 100 um nozzle tip operating at a pressure of 30 psi. To accurately distinguish between GFP signal and autofluorescence, cell suspensions from wild-type GFP^-^ embryos were used for calibration prior to each sorting event. Cells were sorted and 20,000 GFP^+^ cells collected into 300 μL RLT Lysis Buffer (Qiagen, Valencia, CA, USA) containing 10 μL β-mercaptoethanol (Bio-Rad, Life Science Research, Hercules, CA, USA). Control or E3MO sorted cell samples in Lysis Buffer were pooled in groups corresponding to a total of 80,000 to 100,000 cells for total RNA extraction (control groups, n = 5; E3MO groups, n = 3).

### Real time quantitative PCR

Total RNA was extracted from FAC sorted GFP^+^ cells using RNA column-based isolation kits, RNeasy® Micro kit (Qiagen). Concentration and integrity of the RNA extracted from FAC sorted cells were determined with an Agilent 2100 Bioanalyzer and by use of the Agilent RNA 6000 Pico Kit (Agilent Technologies, Santa Clara, CA, USA). The UCAMC Molecular Discovery Core performed the PCR analysis. The extracted RNA was treated with Amplification Grade DNAse (Invitrogen, Life Technologies Corporation, Carlsbad, CA, USA). Super Scripcd VILO™ cDNA Synthesis kit (Invitrogen, Life Technologies Corporation) was used for reverse transcription. The cDNA was enriched using the TaqMan® PreAmp Master Mix kit (Applied Biosystems, Life Technologies Corporation). Proprietary assays with primers and probes were purchased from Life Technologies (*scn8aa*- Dr03093370_m1). Gene expression was measured on ABI’s 7500Fast Instrument using TaqMan® Gene Expression Master Mix (Applied Biosystems, Life Technologies Corporation). The relative standard curve method was used with *scn8aa* expression normalized to *eef1α1a* (DR03119741_g1) [77]. The validity of *eef1α1a* as an endogenous control was tested by measuring the standard deviation of the Ct of all samples at equal concentrations. The standard deviation was consistently below 0.5, indicating non-significant variation of *eef1α1a* between samples. The data were analyzed with the 7500 software, version 2.0.6 from ABI using all default parameters.

### Embryo preparation for electrophysiology

We used previously reported methods to prepare embryos for electrophysiological studies [29,67]. Briefly, zebrafish embryos were mounted using veterinarian suture glue (3 M Vetbond; Revival Animal Health, Orange City, IA, USA) onto a sylgard-coated recording chamber (Dow Corning Corp, Midland, MI, USA), and sacrificed in the presence of 0.01% tricaine (ethyl 3-aminobenzoate methanesulfonate salt; Sigma-Aldrich) prior to trunk skin removal. Embryos were mounted laterally or dorsally to facilitate recordings from ventral or dorsal spinal neurons, respectively. Following extensive washes with Ringer solution (in mM: 145 NaCl, 3 KCl, 1.8 CaCl_2_ and 10 HEPES, pH 7.4) to remove tricaine, embryos were transferred to the appropriate external recording solution. Blunt dissection with polished borosilicate glass electrodes removed muscle and meninges and exposed ventral or dorsal spinal neurons. We focused on neurons that reside within spinal cord segments above the yolk extension (ventral neuron recordings) or yolk sac (dorsal neuron recordings) of 22 to 24 or 24 to 26 hpf embryos, respectively.

### Electrophysiology

Whole-cell current- and voltage-clamp recordings were obtained from ventral and dorsal spinal neurons using patch electrodes (2.5-3.5 MΩ) and an Axopatch-200B amplifier (Molecular Devices, Sunnyvale, CA, USA) as performed previously [29,67]. Electrodes were made using a P-97 microelectrode puller (Sutter Instruments, Novato, CA, USA) and filled with intracellular pipette solution (in mM: 135 KCl, 10 EGTA and 10 HEPES, pH 7.4). We included a fluorescent dye, AlexaFluor 594 (60 to 100 μM; Invitrogen, Eugene, OR, USA) in the pipette solution to label the recorded neuron’s processes for subtype identification and characterization of morphological changes of control versus morphant embryos. Images of neurons filled with the AlexaFluor 594 were captured with an AxioCamHRC camera operating under control of Axiovision 3.0 software (Carl Zeiss, Inc.).

We recorded whole-cell outward and inward currents under voltage-clamp conditions. A P/8 protocol was used for subtraction of passive leak and capacitative transients. Clampex 9.2 (Molecular Devices) was used for data acquisition, and analysis was performed with Clampfit 9.2 (Molecular Devices) and Axograph X (AxoGraph Scientific, Sydney, Australia). Series resistance was compensated by 70 to 80%.

A first series of recordings were done using extracellular solutions that allow flow of all voltage-dependent inward and outward currents, in order to assess currents that contribute to action potential generation. The bath solution consisted of (mM): 125 NaCl, 2 KCl, 10 CaCl_2_ and 5 HEPES, pH 7.4. α-bungarotoxin (0.4 to 0.8 μM; Tocris, Ellisville, MO, USA) was added to the bath solution to immobilize embryos during recordings. Currents were elicited by voltage steps from -40 to 110 mV in 10 mV increments applied from a holding potential of -80 mV. Under these conditions, the recorded outward current represents the net of both voltage and calcium-dependent currents (I_Kv/Ca_). For statistical comparisons, we measured the amplitude of I_Kv/Ca_ at +40 mV, a potential at which the majority of the conductance is activated. We measured the steady-state current during the final 10 ms of the recording.

Similarly, sodium and calcium conductances both contribute to the net inward current (I_Na/Ca_). For I_Na/Ca_, we measured the peak current amplitude, to assess currents when the majority of conductance is activated. Even though I_Na/Ca_ reflects both inward sodium and calcium currents, the peak serves as a good measure of I_Na_, as shown by performing recordings in the presence or absence of 0.1 mM CdCl_2_, a blocker of voltage-dependent calcium currents. The peak inward current amplitude is not affected by inclusion of 0.1 mM CdCl_2_ (no CdCl_2_: -1.6 ± 0.2 nA, n = 13; +CdCl_2_: -1.6 ± 0.4 nA, n = 6).

For the statistical comparisons, we converted current amplitudes to current densities by dividing by cell surface area to normalize current amplitudes to membrane surface area. Cell surface area was computed from the cell’s capacitance, measured in Farads (F), as determined from the capacitative transient for a +10 mV depolarizing step. The standard factor of 1 μF/cm^2^ was used to convert cell capacitance to surface area. Steady-state I_Kv/Ca_ and peak I_Na/Ca_ were normalized to surface area and presented as current densities.

We also recorded voltage-dependent potassium currents (I_Kv_) under conditions of pharmacological and ionic isolation from I_KCa_. To prevent activation of I_KCa_, we blocked calcium currents via substitution of cobalt for calcium in the bath solution (mM: 80 NaCl, 3 KCl, 5 MgCl_2_, 10 CoCl_2_, and 5 HEPES, pH 7.4) accompanied by addition of tetrodotoxin (300 to 500 nM; Calbiochem, Gibbstown, NJ, USA) to block sodium currents. The same pipette solution and voltage steps described above were used for these recordings.

To elicit action potentials and voltage responses to current injection, we recorded from neurons in current-clamp mode using the same bath conditions as described above for whole-cell voltage-clamp recordings of I_Kv/Ca_ and I_Na/Ca_. After measuring the resting membrane potential, we set the membrane potential between -60 and -75 mV by steady current injection. Brief current injections (1 ms), ranging between 0.05 and 0.5 nA, were then applied to elicit single action potentials and determine the minimum amount of current required to elicit an action potential (rheobase). Each stimulus was followed by a 1 second recovery period to avoid sodium channel inactivation. To elicit action potentials from KA” interneurons, hyperpolarizing pulses (100 ms) were applied (-0.05 to 0.05 nA). From recordings of action potentials, we measured several parameters including time and rate of rise (from threshold to peak amplitude), time and rate of decay (from peak to 50% decay), and duration (time between threshold and 50% membrane repolarization).

### Data presentation and statistical analysis

Data are presented as mean ± standard error of the mean (SEM). The properties of CaPs or RBs in uninjected and control morphant embryos were similar and pooled for statistical comparisons. Statistical analysis was performed using Instat software (GraphPad Software, Inc. La Jolla, CA, USA) using unpaired two-tailed *t*-tests or one-way analysis of variance. When multiple comparisons were performed, appropriate *P* value corrections (for example, Bonferroni) were made. Our cut-off for statistical significance was *P* < 0.05; tests yielding statistically significant comparisons are indicated with their *P* values.

## Abbreviations

AHP: afterhyperpolarization; CaP: caudal primary motor neuron; CtlMO: 5-base mismatched *islet1*(Sp)E3MO; CoPA: commissural primary ascending interneurons; CoSA: commissural secondary ascending interneuron; DoLA: dorsal lateral ascending interneuron; Dorsal Comm: dorsal commissural interneuron; DRG: dorsal root ganglion; E3MO: *Islet1*(Sp)E3MO; FAC: fluorescence-activated cell; GABA: γ-aminobutyric acid; GFP: green fluorescent protein; HD: homeodomain; hpf: hours post-fertilization; IC: ipsilateral caudal; KA: Kolmer-Agduhr; Lhx3: LIM homeobox 3; LIM: Lin/Isl/Mec-like; NLI: nuclear LIM interactor; PCR: polymerase chain reaction; PMN: primary motor neuron; pSTAT3: Phosphorylated STAT3; RB: Rohon-Beard cell; VeLD: ventral lateral descending.

## Competing interests

The authors declare that they have no competing interests.

## Authors’ contributions

RLM and ABR conceived of and designed study; RLM performed all experiments; RLM and ABR wrote the manuscript and read and approved the final version.

## Supplementary Material

Additional file 1 **Identification of ventral interneurons for electrophysiological study.** (**A** and **A’**) VeLDs were identified *in situ* in 24 hpf *Tg(mnx1:gfp)ml2* embryos. **(A)** VeLD (red arrow) has a characteristic position slightly rostral to CaP (white arrow) and also expresses GFP in the *Tg(mnx1:gfp)ml2* line. The CaP motor axon (asterisk) projects ventrally. **(A’)** The fluorescence image of Panel A is superimposed on the bright field image. Scale Bar = 50 μm in **A** for **A** and **A’**. (**B** to **C’**) KA” and KA’ interneurons were identified i*n situ* in 24 hpf *Tg(8.1kGata1:eGFP)* embryos. **(B)** KA”s have a ventral location and extend an axon (asterisk) rostrally. **(B’)** The fluorescence image of Panel B is superimposed on the bright field image. **(C)** KA’s reside slightly more dorsal than do KA”s and also extend an axon (asterisk) rostrally. **(C’)** The fluorescence image of Panel **C** is superimposed on the bright field image. Scale Bar = 50 μm in **B** for **B** to **C’**. Click here for file

Additional file 2 **E3MO prevents processing of *isl1 *** **mRNA and knocks-down protein expression.** We used assays developed by Hutchinson and Eisen [13] to demonstrate the efficacy of the E3MO. (**A** and **B**) RNA *in situ* hybridization for *isl1* mRNA shows cytoplasmic localization in Ctl **(A)** and nuclear retention in E3 morphant (B) embryos. Scale Bar = 50 μm in **B** for **A** and **B**. (**C** and **D**) Islet1/2 immunoreactivity is present dorsally and ventrally in Ctl **(C)** but substantially reduced ventrally and to a lesser extent dorsally in E3 morphant **(D)** embryos. Scale Bar = 50 μm in **D** for **C** and **D**. Click here for file
