# Supplementary figures and images for: Spinal neurons require Islet1 for subtype-specific differentiation of electrical excitability
Source: Neural Dev. 2014 Aug 22;9:19. doi: 10.1186/1749-8104-9-19 (PMC4153448; doi:10.1186/1749-8104-9-19)

*Tg(mnx1:gfp)*

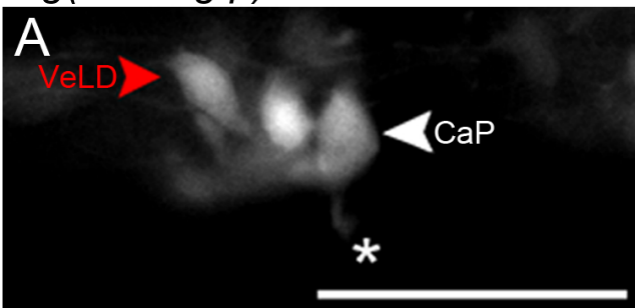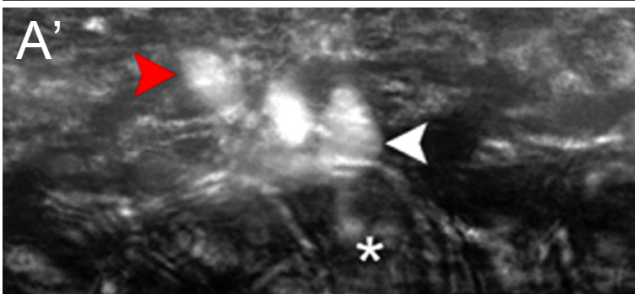

*Tg(8.1kGata1:eGFP)*

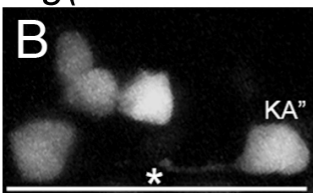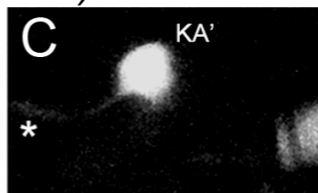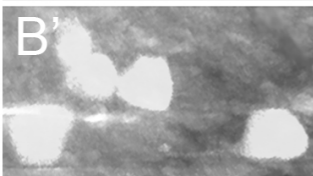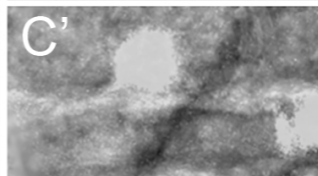

Supplement: Additional file 1 — Identification of ventral interneurons for electrophysiological study. (A and A’) VeLDs were identified in situ in 24 hpf Tg(mnx1:gfp)ml2 embryos. (A) VeLD (red arrow) has a characteristic position slightly rostral to CaP (white arrow) and also expresses GFP in the Tg(mnx1:gfp)ml2 line. The CaP motor axon (asterisk) projects ventrally. (A’) The fluorescence image of Panel A is superimposed on the bright field image. Scale Bar = 50 μm in A for A and A’. (B to C’) KA” and KA’ interneurons were identified in situ in 24 hpf Tg(8.1kGata1:eGFP) embryos. (B) KA”s have a ventral location and extend an axon (asterisk) rostrally. (B’) The fluorescence image of Panel B is superimposed on the bright field image. (C) KA’s reside slightly more dorsal than do KA”s and also extend an axon (asterisk) rostrally. (C’) The fluorescence image of Panel C is superimposed on the bright field image. Scale Bar = 50 μm in B for B to C’. [file 1749-8104-9-19-S1.pdf]

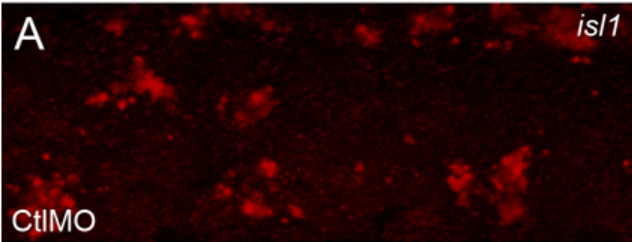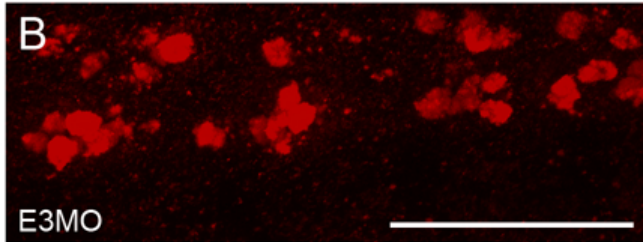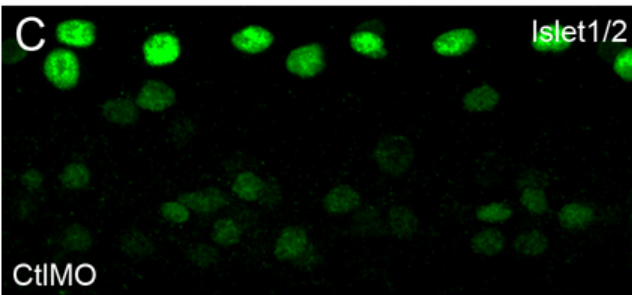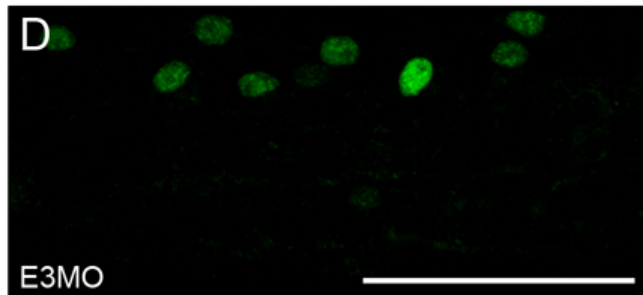

Supplement: Additional file 2 — E3MO prevents processing of isl1 mRNA and knocks-down protein expression. We used assays developed by Hutchinson and Eisen [13] to demonstrate the efficacy of the E3MO. (A and B) RNA in situ hybridization for isl1 mRNA shows cytoplasmic localization in Ctl (A) and nuclear retention in E3 morphant (B) embryos. Scale Bar = 50 μm in B for A and B. (C and D) Islet1/2 immunoreactivity is present dorsally and ventrally in Ctl (C) but substantially reduced ventrally and to a lesser extent dorsally in E3 morphant (D) embryos. Scale Bar = 50 μm in D for C and D. [file 1749-8104-9-19-S2.pdf]
